# Prevalence of *BRCA1* Mutations in Familial and Sporadic Greek Ovarian Cancer Cases

**DOI:** 10.1371/journal.pone.0058182

**Published:** 2013-03-11

**Authors:** Alexandra V. Stavropoulou, Florentia Fostira, Maroulio Pertesi, Marianthi Tsitlaidou, Gerassimos E. Voutsinas, Olga Triantafyllidou, Aristotelis Bamias, Meletios A. Dimopoulos, Eleni Timotheadou, Dimitrios Pectasides, Christos Christodoulou, George Klouvas, Christos Papadimitriou, Thomas Makatsoris, George Pentheroudakis, Gerasimos Aravantinos, Vassilis Karydakis, Drakoulis Yannoukakos, George Fountzilas, Irene Konstantopoulou

**Affiliations:** 1 Molecular Diagnostics Laboratory, INRaSTES, National Center for Scientific Research “Demokritos”, Athens, Greece; 2 Laboratory of Environmental Mutagenesis and Carcinogenesis, Institute of Biosciences and Applications, National Center for Scientific Research “Demokritos”, Athens, Greece; 3 Department of Clinical Therapeutics, Alexandra Hospital, University of Athens School of Medicine, Athens, Greece; 4 Department of Medical Oncology, Papageorgiou Hospital, Aristotle University of Thessaloniki School of Medicine, Thessaloniki, Greece; 5 Oncology Section, Second Department of Internal Medicine, “Hippokration” Hospital, Athens, Greece; 6 Second Department of Medical Oncology, “Metropolitan” Hospital, Piraeus, Greece; 7 Division of Oncology, Department of Medicine, University Hospital, University of Patras Medical School, Patras, Greece; 8 Department of Medical Oncology, Ioannina University Hospital, Ioannina, Greece; 9 Second Department of Medical Oncology, “Agii Anargiri” Cancer Hospital, Athens, Greece; 10 1st Department of Surgery, Rhodes General Hospital, Rhodes, Greece; IFOM, Fondazione Istituto FIRC di Oncologia Molecolare, Italy

## Abstract

Germline mutations in the *BRCA1* and *BRCA2* genes contribute to approximately 18% of hereditary ovarian cancers conferring an estimated lifetime risk from 15% to 50%. A variable incidence of mutations has been reported for these genes in ovarian cancer cases from different populations. In Greece, six mutations in *BRCA1* account for 63% of all mutations detected in both *BRCA1* and *BRCA2* genes. This study aimed to determine the prevalence of *BRCA1* mutations in a Greek cohort of 106 familial ovarian cancer patients that had strong family history or metachronous breast cancer and 592 sporadic ovarian cancer cases. All 698 patients were screened for the six recurrent Greek mutations (including founder mutations c.5266dupC, p.G1738R and the three large deletions of exon 20, exons 23–24 and exon 24). In familial cases, the *BRCA1* gene was consequently screened for exons 5, 11, 12, 20, 21, 22, 23, 24. A deleterious *BRCA1* mutation was found in 43/106 (40.6%) of familial cancer cases and in 27/592 (4.6%) of sporadic cases. The variant of unknown clinical significance p.V1833M was identified in 9/698 patients (1.3%). The majority of *BRCA1* carriers (71.2%) presented a high-grade serous phenotype. Identifying a mutation in the *BRCA1* gene among breast and/or ovarian cancer families is important, as it enables carriers to take preventive measures. All ovarian cancer patients with a serous phenotype should be considered for genetic testing. Further studies are warranted to determine the prevalence of mutations in the rest of the *BRCA1* gene, in the *BRCA2* gene, and other novel predisposing genes for breast and ovarian cancer.

## Introduction

Ovarian cancer is one of the highest mortality rated malignancies, an aspect mainly attributed to the advanced stage at diagnosis. Although new predisposing genes have been identified lately, the important players to ovarian cancer susceptibility are still the known *BRCA1* and *BRCA2* genes. Carriers of inherited mutations in *BRCA1* and *BRCA2* genes face a lifetime risk of ovarian cancer of 35–60% (average age of diagnosis 50 years) and 12–25% (average age of diagnosis 60 years) respectively, and also an elevated risk of fallopian tube and peritoneal carcinomas [1]–[3].

*BRCA1* and *BRCA2-*associated ovarian malignancies have a distinct clinical phenotype, the majority of which being high-grade serous, advanced stage carcinomas [4], while generally are being associated with overall longer survival [5], [6]. Moreover, the *BRCA1/2* mutation status of an ovarian cancer patient can be an important aspect in regards to the decision of chemotherapy; *BRCA1/2* carriers show increased sensitivity to platinum-based therapy [6], [7], as well as to poly-ADP-ribose polymerase inhibitors [8].

Hereditary ovarian cancer can also occur in the context of Lynch syndrome, which is caused by inherited germline mutations within the MMR genes. The cumulative risk of ovarian cancer in MMR mutation carriers is estimated to be 10%, while histologically these tumours are of the endometrioid subtype in most cases [9].

Hereditary ovarian cancer is probably underestimated, since recent studies highlight new susceptibility genes (*RAD51C, RAD51D, PALB2*) that might predispose for ovarian cancer, but their exact prevalence is still under investigation [10]–[13]. Up-to date sequencing technologies that provide the opportunity to test massively multiple targeted genes have been already applied in ovarian cancer genetics. The most interesting is the test based on BROCA chip, a highly sensitive panel of 21 tumor suppressor genes, which tested 360 ovarian cancer cases and successfully identified deleterious mutations in 12 known ovarian susceptibility genes, with the substantial proportion represented by *BRCA1* and *BRCA2* mutations [13].

The prevalence of *BRCA1* and *BRCA2* mutations in ovarian cancer patients varies amongst populations; a quite thorough population study in North America demonstrates a 13–15% frequency of germline *BRCA1/2* mutations in sporadic ovarian cases [14], [15]. The prevalence of mutations can rise up to 30–40% in populations such as Ashkenazi Jews, where a number of founder mutations are apparent [16].

Although the Greek population is characterized by genetic heterogeneity, our extensive 15–year research on hereditary breast/ovarian cancer has highlighted the existence of 6 recurrent mutations including four founder (c.5266dupC, p.G1738R, delex20,delex24) accounting for 63% of all mutations identified in *BRCA1/2* genes and 73% of mutations identified in *BRCA1* only [17]–[21]. This bipolar project focuses on the prevalence of *BRCA1* mutations among 106 familial and 592 sporadic Greek ovarian cancer cases with the simultaneous correlation of clinicopathological tumour features.

## Materials and Methods

### Patient Study Group

The study group consisted of patients with epithelial ovarian cancer that were recruited from various hospitals around Greece in collaboration with the Hellenic Cooperative Oncology Group (HeCOG) between 2000 and 2012. Corresponding demographic, clinicopathological data had been registered for the majority of recruited patients in the frame of clinical service in HeCOG-affiliated hospitals. Samples from 698 patients in total, selected on the basis of a diagnosis of ovarian carcinoma, were analyzed for mutations in *BRCA1*. The 698 patients were categorized as: (a) Familial cases: 106 ovarian cancer patients with at least one first degree relative presented with either breast and/or ovarian cancer or cancer patients presented with breast and ovarian cancer. 53/106 (50%) patients had both breast and ovarian cancer, 29 of which had family history and 24 with no family history. The mean age of diagnosis was 49 years, ranging from 24 to 72 years old, and (b) sporadic cases: 592 ovarian cancer cases were included based on their diagnosis of ovarian cancer only and had no reported family history. The mean age of diagnosis was 59.1 years, ranging from 17 to 82 years old.

All patients had signed an informed consent form permitting the use of their biologic material for research purposes and a research nurse explained the purpose of the study to them; the possibility of increased risk of ovarian cancer (and possibly other cancers) in family members were explained in detail. Patients donated 10 ml of blood for genetic analysis as well as completed a questionnaire containing details on other cancers in family members. In cases where a mutation was identified, diagnosis and family history was confirmed and segregation analysis was performed. Furthermore, family relatives of mutation carriers were informed in specialized counselling sessions and if consented to genetic analysis, they were tested for the specific mutation. Histology report, where available, was provided by the originating hospital, from which information regarding tumour type, grading and staging was extracted. The study was approved by the Bioethics Committees of the IRB of Papageorgiou Hospital of Thessaloniki and NCSR “Demokritos”.

### Mutation Analysis

Total genomic DNA was isolated from peripheral blood lymphocytes following the salt extraction procedure. DNA quantitation was assessed by UV absorbance using a Nanodrop™ ND-1000 spectrophotometer (Thermo Fisher Scientific, MA, USA). *BRCA1* (NM_007294.3) mutation screening was based on a population-specific hierarchical protocol described previously (18), aiming to reduce time and cost involved. Two different experimental protocols were designed: For sporadic cases, screening was performed only for the six most common *BRCA1* mutations identified in the Greek population. Exon 20 of the *BRCA1* gene (encompassing the founder mutations: p.G1738R & c.5266dupC and the recurrent p.R1751X mutation) was analyzed by direct sequencing, while the three Greek founder genomic rearrangements involving exons 20, 23 and 24 were assessed by three individual diagnostic PCRs [20] (Pertesi et al. in preparation). Additionally, in familial cases full sequencing of exons 5, 11, 12, 20, 21, 22, 23, 24 (which cover 70% of the gene’s coding sequence) was performed. These regions contain all *BRCA1* mutations previously identified in the Greek population. All PCR amplifications were performed in a Veriti 96-Well Thermal Cycler (Applied Biosystems, Foster City, CA). PCR product purification was performed using a vacuum driven ultrafiltration purification system, where PCR samples are transferred to a filter plate with a membrane resin which retains the PCR products free from non-incorporated nucleotides and primers (Macherey-Nagel, Düren, Germany). Sequencing reactions were performed using the v.3.1 BigDye Terminator Cycle Sequencing kit (Applied Biosystems, Foster City, CA) and PCR products were electrophoresed on the ABI Prism® 3130xl Genetic Analyzer. Sequences obtained were aligned, using Sequencher® PC software (Gene Codes, USA), with reference sequences from Genbank (NG_005905.2) and examined for the presence of mutations. Upon mutation identification, an independent blood sample was drawn from the patient and the mutation was confirmed by bi-directional sequencing. In addition statistical analysis was performed using the chi-square test.

### Real-Time PCR (Allele Discrimination)

Allele discrimination of the c.5497G>A (p.V1833M) variant in the sporadic cohort was performed by TaqMan® assay using a Mx3000P™ Real-Time PCR System (Stratagene, USA) and analyzed using the allelic discrimination endpoint analysis with data collected at the end of the PCR process. Each reaction included a primer pair used to amplify the 106 bp product and two fluorescent probes, labelled with two spectrally distinct dyes, namely FAM (G) and HEX (A), allowing genotyping of the two possible variants at the single-nucleotide polymorphism (SNP) site in a target template sequence. The KAPA PROBE FAST Universal qPCR Master mix kit (KapaBiosystems) was used, containing all components except primers and probes. ROX reference dye was additionally added in each reaction mix. PCR amplification was performed in a 20 µl reaction using 50 ng genomic DNA, and a fast-2-step cycling protocol with the following conditions: enzyme activation at 95°C for 3 min, and 40 cycles of denaturation at 95°C for 3 sec and annealing/extension at 60°C for 15 sec. Post-run analysis determined either a normal homozygote (samples having both alleles normal) or heterozygote (samples having normal allele as well as the *BRCA1* allelic variant c.5497G>A). The Ct value of an allele-specific probe highlights the presence of the examined SNP. Heterozygotes were confirmed by direct DNA sequencing. Threshold fluorescence (used to determine Ct values) was adjusted by baseline-corrected normalized fluorescence (dRn). Probe and primer sequences used are available from the authors upon request.

## Results

**Figure 1:**
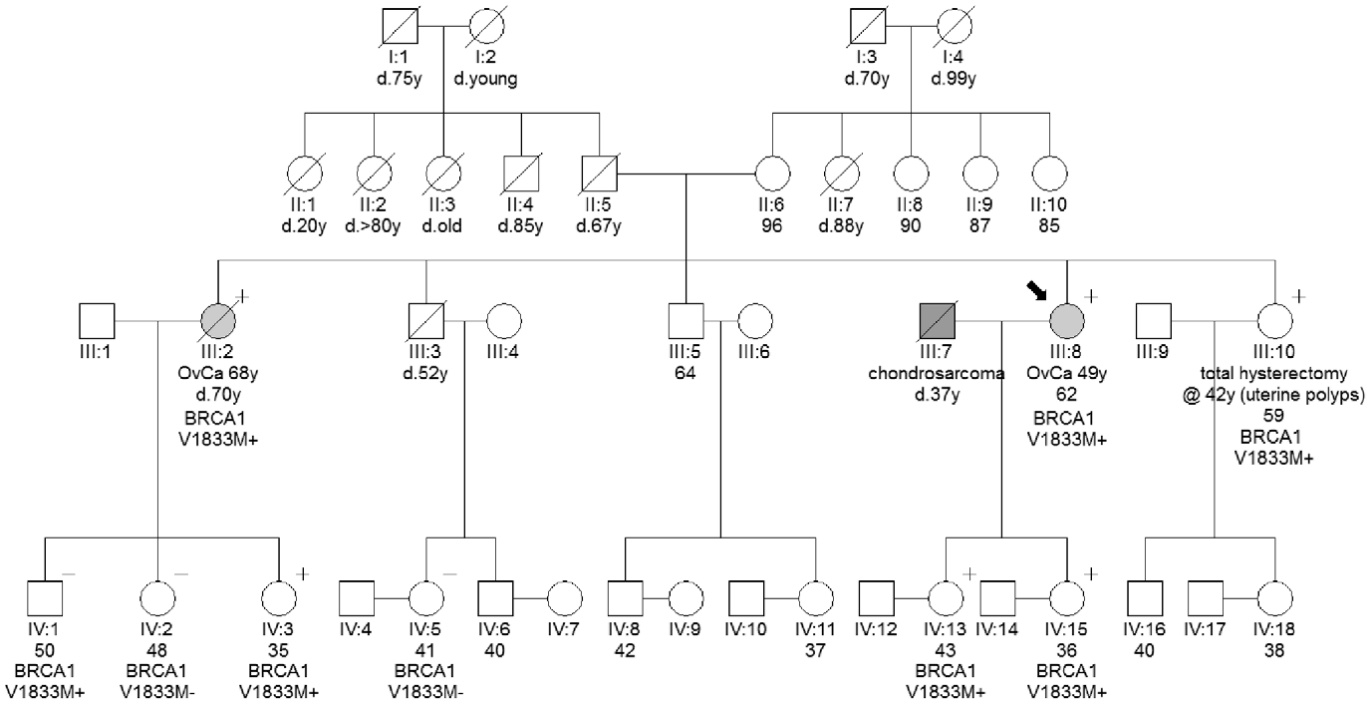
Pedigree of a family with the *BRCA1* p.V1833M variant of uncertain significance (VUS). Pink circles indicate women with ovarian cancer. The plus sign indicates the presence of the variant in the indicated person, while the minus sign indicates the absence of the variant. OvCa = ovarian cancer.

106 familial and 592 sporadic ovarian cancer cases were analyzed to determine the frequency of the six most common mutations of the *BRCA1* gene in the Greek population, namely c.5266dupC, p.Gly1738Arg, p.Arg1751X, deletion of exon 20 (c.5256_5277+3179del3200), deletion of exon 24 (c.5468-285_5592+4019del4429_insCACAG) and deletion of both exons 23 and 24 (g.169527_180579del11052). Considering the mutation prevalence of only the above six recurrent mutations, there is a substantial difference between familial (25.5%, 27/106) and sporadic cases (4.4%, 26/592) (p-value <0.0001). Familial cases were further screened for mutations in all *BRCA1* exons where a mutation was previously identified in the Greek population (exons 5, 11, 12, 20, 21, 22, 23, 24), increasing mutation prevalence in this group to 40.6% (43/106). All mutations found, together with carriers’ age of onset and histology, are shown in Table 1 for familial cases and in Table 2 for sporadic cases. Interestingly, an extremely rare variant of unknown clinical significance (VUS), *BRCA1* p.V1833M (rs80357268), was found in 9 patients (1.3%) out of the 698 in total. Prevalence in the sporadic group, screened by real-time PCR, was 0.85% (5/592), while 3.7% (4/106) in the familial group, screened by direct sequencing of exon 24 (Table 3). Interestingly, all familial patients that carried this specific variant had a family history of breast and/or ovarian cancer. Segregation analysis done in one of the families tested positive showed that the p.V1833M variant co-segregated with the disease, suggesting a possible deleterious effect (Fig. 1). A broad cross-validation, based on biochemical and cell-based transcriptional assay, of BRCT missense variants highlighted the severe folding defect and the compromised effect on transcription of the p.V1833M variant [22]. Due to the scarcity of this variant no other segregation data were found in literature, however all available *in vitro* and *in silico* evaluation data agree upon its classification as deleterious [23]–[25]. In addition, no allele frequency was observed in dbSNP. In the present study, carriers of this variant are not included in our results as positive for a mutation, as we feel more solid clinical and functional data are needed to confirm its pathogenicity.

**Table 1:** *BRCA1* pathogenic mutations identified in familial cases.

FAMILIAL CASES									
					Histologic diagnosis				
Patient	Exon	Mutation (cDNA)	Mutation (protein)	Age of onset	Type	Grade	Stage	Other primary cancers in proband (Age)	Family history (including proband)
406	5	c.181T>G	p.Cys61Gly	39	serous papillary adenocarcinoma	ΙΙΙ	Ιc	_	1 BrCa <40y & 1 OvCa
1126β	5	c.181T>G	p.Cys61Gly	52	N.A.	N.A.	N.A.	_	3 BrCa (1 BrCa <40y),1 OvCa
98	11	c.1504_1508delΤΤΑΑΑ	p.Leu502fs	44	serous papillary cystadenocarcinoma	N.A.	IV	_	1 BrCa <30y & 1 OvCa
99	11	c.2980delT	p.Cys994fs	33	serous cystadenocarcinoma	N.A.	IIIc	_	3 OvCa & 1 BrCa+OvCa
145	11	c.3158_3159insG	p.Val1054fs	72	serous	N.A.	N.A.	BrCa (62y)	1 BrCa+OvCa & 2 OvCa
699	11	c.3700_3704delGTAAA	p.Val1234fs	45	serous	III	N.A.	BrCa (42y & 43y)	4 BrCa <45y & 3 OvCa
657	11	c.3700_3704delGTAAA	p.Val1234fs	48	serous papillary cystadenocarcinoma	III	IV	BrCa (50y)	1 BrCa+OvCa
483β	11	c.3375_3376delTC	p.Ser1125fs	38	N.A.	N.A.	N.A.	_	1 BrCa <30y +1 OvCa
211	11	c.3779delT	p.Leu1260fs	48	serous papillary adenocarcinoma	N.A.	N.A.	BrCa (48y)	1 BrCa+OvCa
230	11	c.3607C>T	p.Arg1203X	46	papillary serous	II–III	N.A.	BrCa 26y & bilateral BrCa 40y	4 BrCa <40y & 2 OvCa
1152	11	c.1505T>G	p.Leu502x	51	bilateral serous	III	N.A.	_	2 OvCa & 2 BrCa
1181	11	c.1210insCT	p.Glu404fs	50	N.A.	N.A.	N.A.	BrCa (39y)	1 BrCa <40y & 2 OvCa
870	11	c.2648insGGCA	p.Ala883fs	44	serous	N.A.	N.A.	_	2 OvCa <45y
824β	12	c.4168_4169 delTG	p.Ser1389fs	57	N.A.	N.A.	N.A.	_	1 BrCa <35y & 2 OvCa
1077	19	c.5161A>C	p.Gln1721Pro*	35	bilateral serous	ΙΙΙ	N.A.	_	2 OvCa
272	20	c.5194-452_5277+3638del4174	p.His1732_Lys1759del	52	serous cystadenocarcinoma	II–III	N.A.	BrCa (40y)	1 BrCa+OvCa
398	20	c.5212G>A	p.Gly1738Arg	57	adenocarcinoma	N.A.	IV	_	2 OvCa
798	20	c.5212G>A	p.Gly1738Arg	41	adenocarcinoma	N.A.	N.A.	BrCa (39y)	1 BrCa+OvCa
287α	20	c.5212G>A	p.Gly1738Arg	32	N.A.	N.A.	N.A.	_	1 BrCa <50y & 2 OvCa
294	20	c.5212G>A	p.Gly1738Arg	68	serous cystadenocarcinoma	ΙΙΙ	N.A.	BrCa (72y)	1 BrCa+OvCa
439	20	c.5251C>T	p.Arg1751X	48	N.A.	N.A.	N.A.	_	6 BrCa (1 BrCa+OvCa) & 2 OvCa
290	20	c.5251C>T	p.Arg1751X	61	serous adenocarcinoma	N.A.	III	BrCa (66y)	1 BrCa+OvCa
298	20	c.5251C>T	p.Arg1751X	40	serous papillary cystadenocarcinoma	III	IV	BrCa (30y)	3 BrCa <50y (1 BrCa+OvCa <40y)
189	20	c.5251C>T	p.Arg1751X	42	serous	III	N.A.	BrCa (41y)	2 BrCa <40y (1 BrCa+OvCa)
1024	20	c.5256_5277+3179del3200	p.Arg1753fs	48	serous papillary cystadenocarcinoma	III	N.A.	_	3 BrCa (1 BrCa <50y) & 1 OvCa (1 BrCa+OvCa)
428	20	c.5256_5277+3179del3200	p.Arg1753fs	56	N.A.	N.A.	N.A.	BrCa (58y)	1 BrCa+OvCa & 1 BrCa
698	20	c.5256_5277+3179del3200	p.Arg1753fs	48	N.A.	N.A.	N.A.	BrCa (38y & 42y)	4 BrCa (3 BrCa <45y) & 2 OvCa
296	20	c.5266dupC	p.Gln1756fs	56	serous cystadenocarcinoma	ΙΙ	IV	BrCa (48y)	1 BrCa+OvCa
280	20	c.5266dupC	p.Gln1756fs	45	N.A.	N.A.	N.A.	_	3 OvCa
1186	20	c.5266dupC	p.Gln1756fs	49	serous papillary adenocarcinoma	III	III	_	1 BrCa <30y & 3 OvCa
520	20	c.5266dupC	p.Gln1756fs	35	bilateral endometrioid carcinoma	N.A.	III	_	1 bil OvCa
344	20	c.5266dupC	p.Gln1756fs	50	serous cyst-adenocarcinoma	ΙΙΙ	N.A.	BrCa (72y)	2 BrCa (1 BrCa <40y) & 1 OvCa
365α	20	c.5266dupC	p.Gln1756fs	54	clear cell	III	N.A.	_	1 BrCa <35y & 2 OvCa
155	23	c.5467G>A	p.1803_1822del20	50	serous papillary cystadenocarcinoma	I	III	BrCa (40y)	2 BrCa (1 BrCa+OvCa)
159	23	c.5434C>G	p.Pro1812Αla**	61	N.A.	N.A.	N.A.	BrCa	1 BrCa+OvCa
284	24	c.5468-285_5592+4019del4429_insCACAG	p.0	52	endometrioid carcinoma	N.A.	N.A.	BrCa (53y)	2 BrCa (1 BrCa+OvCa)
1174	24	c.5468-285_5592+4019del4429_insCACAG	p.0	53	serous papillary cyst-adenocarcinoma	II	N.A.	BrCa (61y)	1 BrCa+OvCa, 5 BrCa
332	24	c.5468-285_5592+4019del4429_insCACAG	p.0	31	bilateral serous cyst-adenocarcinoma	N.A.	N.A.	BrCa (28 y)	1 BrCa+OvCa
440	24	c.5468-285_5592+4019del4429_insCACAG	p.0	48	undifferentiated carcinoma	N.A.	N.A.	BrCa (69y)	1 BrCa+OvCa
469	24	c.5468-285_5592+4019del4429_insCACAG	p.0	51	serous surface papillary	III	IV	_	1 BrCa & 1 OvCa
416	23,24	g.169527_180579del11052	p.Gly1803_Tyr1863del11052	39	serous papillary adenocarcinoma	N.A.	N.A.	BrCa (37y)	1 BrCa+OvCa
1036	23,24	g.169527_180579del11052	p.Gly1803_Tyr1863del11052	51	serous	ΙΙΙ	N.A.	BrCa (51y)	3 BrCa (1 BrCa <50y) & 2 OvCa (1 BrCa+OvCa)
661	23,24	g.169527_180579del11052	p.Gly1803_Tyr1863del11052	67	N.A.	N.A.	N.A.	BrCa (47y & 60y)	5 BrCa & 2 OvCa
1247	23,24	g.169527_180579del11052	p.Gly1803_Tyr1863del11052	36	serous papillary adenocarcinoma	III	III	_	3 OvCa (3 OvCa <35y)

* Unclassified variant.

** Splicing variant causing exon 23 skipping [47].

N.A. Non Applicable.

**Table 2:** *BRCA1* pathogenic mutations identified in sporadic cases.

SPORADIC CASES						
				Histologic diagnosis		
Patient	Exon	Mutation	Age of onset	Type	Grade	Stage
944	5	p.C61G	59	serous papillary cystadenocarcinoma	III	N.A.
399	20	p.G1738R	61	endometrioid	ΙΙΙ	N.A.
915	20	p.G1738R	67	serous papillary cystadenocarcinoma	II	IV
1129	20	p.G1738R	51	adenocarcinoma	III	N.A.
1079	20	p.G1738R	53	serous papillary	ΙΙΙ	N.A.
952	20	p.R1751X	55	clear cell mesonephroid	III	IV
737	20	p.R1751X	69	serous papillary cystadenocarcinoma	N.A.	IV
1205	20	c.5266dupC	56	serous papillary cystadenocarcinoma	ΙΙΙ	N.A.
899	20	c.5266dupC	51	endometrioid	III	IV
1078	20	c.5266dupC	63	endometrioid	III	IV
709α	20	c.5266dupC	65	N.A	N.A	N.A
1080	20	c.5266dupC	58	serous	ΙΙΙ	N.A.
946	20	c.5266dupC	57	endometrioid adenocarcinoma	III	IV
951	20	c.5266dupC	39	serous papillary cystadenocarcinoma	III	IIIc
521	20	c.5266dupC	40	serous cystadenocarcinoma	N.A.	IV
954	20	c.5266dupC	45	serous papillary cystadenocarcinoma	III	IIb
955	20	c.5266dupC	55	serous papillary cystadenocarcinoma	I	IIIc
900	20	c.5256_5277+3179del3200	65	serous papillary cystadenocarcinoma	III	IV
1121	24	c.5468-285_5592+4019del4429_insCACAG	46	endometrioid	ΙΙΙ	N.A.
1130	24	c.5468-285_5592+4019del4429_insCACAG	49	serous papillary cystadenocarcinoma	ΙΙΙ	N.A.
893	24	c.5468-285_5592+4019del4429_insCACAG	50	serous papillary cystadenocarcinoma	III	IV
896	24	c.5468-285_5592+4019del4429_insCACAG	59	serous papillary cystadenocarcinoma	III	IIIa
1055	23,24	g.169527_180579del11052	68	adenocarcinoma	III	IV
1056	23,24	g.169527_180579del11052	39	mucinous cystadenocarcinoma	ΙΙ	IIIc
1057	23,24	g.169527_180579del11052	49	serous papillary cystadenocarcinoma	ΙΙΙ	IIIc
1058	23,24	g.169527_180579del11052	59	serous	N.A.	N.A.
1214	23,24	g.169527_180579del11052	34	undifferentiated carcinoma	N.A.	N.A.

**Table 3:** Showing the characteristics of patients carrying the *BRCA1* p.V1833M variant.

				Histologic diagnosis			
Patient	Exon	Mutation	Age of onset	Type	Grade	Stage	Family history(including proband)
426	24	p.V1833M	62		N.A	N.A.	N.A.
2043	24	p.V1833M	48	serous papillarycyst-adenocarcinoma	III	N.A.	N.A.
2118	24	p.V1833M	48	serous papillarycyst-adenocarcinoma	II	N.A.	N.A.
3249	24	p.V1833M	71	Endometrioid	III	N.A.	N.A.
3275	24	p.V1833M	69	Serous	II	N.A.	N.A.
123	24	p.V1833M	59	Endometrioid	III	N.A.	2 OvCa
301	24	p.V1833M	45	N.A.	N.A.	N.A.	2 OvCa & 1 BrCa <50y
460	24	p.V1833M	63	serous papillarycyst-adenocarcinoma	N.A.	IV	2 BrCa >50y & 1 OvCa
791	24	p.V1833M	49	serous adenocarcinoma	III	Ia	2 OvCa

**Figure 2:**
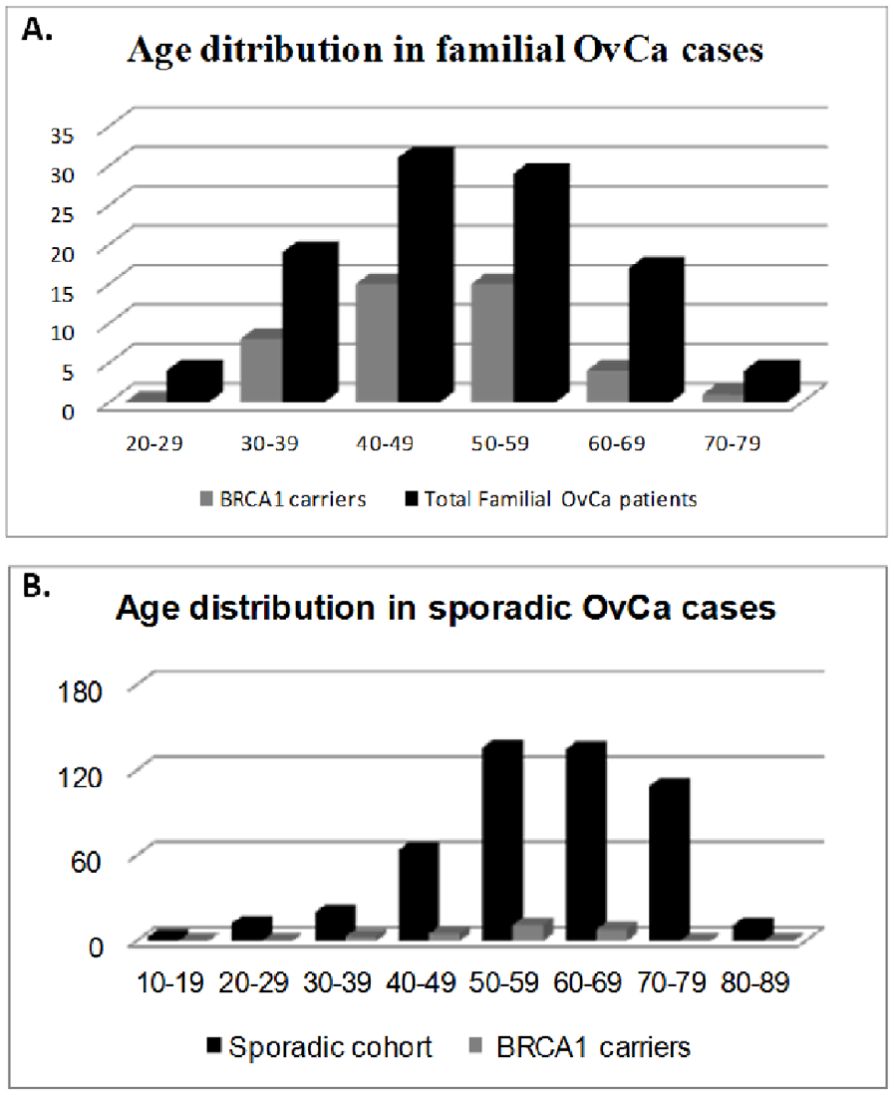
Age of onset distribution in familial and sporadic cases. A) Age of onset distribution among the total of 106 familial patients with ovarian cancer and 41 patients with a *BRCA1* mutation. B) Age of onset distribution among 492/592 of sporadic patients and 27/27 patients with a *BRCA1* mutation.

The age of onset distribution in *BRCA1* carriers compared to total familial cases, as well as for *BRCA1* carriers compared to the total sporadic cases is shown in Fig. 2. Mean age of onset in *BRCA1* carriers in the familial cohort was 48.5 years, whereas in the total group was 49 years, with a range of 24–72 years. In the sporadic group, mean age of onset was 54.2 years for carriers and 59.1 years in the total group, with a range of 17–82 years. Among the 106 familial patients tested, 53 have developed both breast and ovarian cancer (50%). Of the 29 patients who developed both breast and ovarian cancer and had a family history 14/29 (48.3%) carried a *BRCA1* mutation, whereas from those that had no clear family history 11/24 (45.8%) carried a *BRCA1* mutation. However, there is no significant difference between the two groups (p-value 0.8593). Also, the majority of the familial (72.6%) and sporadic (64.6%) cohorts comprised of the serous type of ovarian carcinomas. Correlation between carriers and the total groups of both familial and sporadic cases showed that in accordance with other studies most of the *BRCA1* carriers were of the serous type and there is no statistically significant difference between the two groups (p-value 0.1022). Serous histology presented in 78.8% of the familial *BRCA1* carriers (Table 4) and in 61.5% of the *BRCA1* carriers in the sporadic cohort (Table 5).

**Table 4:** Tumour histology in familial ovarian cancer cases compared to that of *BRCA1* carriers in each group. Histological type was known for 33/43 *BRCA1* carriers and 62/106 in total.

FAMILIAL CASES		
Tumour histology	*BRCA1* carriers	Total No. of cases
Serous	26 (78.8%)	45 (72.6%)
Mucinous	0	0
Clear cell	1 (3%)	1 (1,6%)
Endometrioid	2 (6.9%)	8 (13.8%)
Undifferentiated	1 (3.45%)	1 (1.72%)
Adenocarcinoma	3 (10.3%)	6 (10.3%)
Mixed	0	0
other	0	1 (1.72%)

**Table 5:** Tumour histology in sporadic ovarian cancer cases compared to that of *BRCA1* carriers in each group. Histological type was known for 26/27 *BRCA1* carriers and 450/592 in total.

SPORADIC CASES		
Tumour histology	*BRCA1* carriers	Total No. of Cases
Serous	16 (61.5%)	291 (64.6%)
Mucinous	1 (3.8%)	15 (3.3%)
Clear cell	1 (3.8%)	24 (5.3%)
Endometrioid	5 (19.2%)	44 (9.7%)
Undifferentiated	1 (3.8%)	7 (1.5%)
Adenocarcinoma	2 (7.7%)	21 (4.6%)
Mixed	0	7 (1.5%)
other	0	41 (9.1%)

## Discussion

In this study we have established the prevalence of *BRCA1* mutations in a group of 698 Greek ovarian cancer patients, 106 of which were familial cases and 592 apparently sporadic. We identified 70 pathogenic mutations in all (10%), 43 in the familial cohort (40.6%) and 27 in the sporadic cohort (4.6%). To our knowledge, this is the first such study in the Greek population. A number of similar studies in other populations have been published, giving prevalence for *BRCA1* mutations in ovarian cancer patients from 8 to 11%. Variation could be due to a number of reasons, mainly experimental design and populations with different degrees of genetic heterogeneity. A Canadian study of 1342 ovarian cancer cases revealed a combined mutation frequency of 13.3% of both *BRCA1* and *BRCA2,* 8% of which was *BRCA1* [14]. In another study 11.5% of all ovarian cancer cases in Colombia were attributable to a single *BRCA1* founder mutation, while 15.6% of the total cohort was positive for mutations in either *BRCA1* or *BRCA2* [26]. 5.8% of a population-based series of ovarian cancer cases in Denmark were also found to be positive for *BRCA1/2* mutations [27]. Of 209 women in the Tampa Bay area with invasive ovarian carcinoma, 15.3% had mutations in *BRCA1* or *BRCA2,* 9.5% of which was due to *BRCA1* mutations [15]. Similarly, in a recent Australian study, 8.8% (88/1001) of ovarian cancer patients tested had a *BRCA1* mutation [28]. In a Polish study, *BRCA1* or *BRCA2* germline mutations were found in 13.9% of consecutive ovarian cancer patients, 11% of which was attributable to *BRCA1* [29]. In a Swedish study, 13/161 (8%) of the patients were found to carry a *BRCA1* or *BRCA2* mutation, with 12/13 cases being *BRCA1*-positive [30].

Finally, two pioneer studies using next-generation sequencing techniques [13], [31] reported an 18% prevalence in *BRCA1 & BRCA2* (11.3% for *BRCA1*) mutations in ovarian cancer patients, while the Cancer Genome Atlas (TCGA) Research Network reported 14% (8.5% for *BRCA1*) [32].

The frequency of 10% for *BRCA1* mutations found in the present study in all patients screened, regardless of family history, is consistent with previous observations in other populations. Given the fact that not the entire *BRCA1* gene was screened, this percentage is probably an underestimate of the true frequency in ovarian cancer patients in our population. We estimate that the true frequency could be up to 20% higher, as in 85% of our cohort (592/698 patients) screening included only the Greek founder or recurrent mutations, representing 73% of all *BRCA1* mutations observed in this population [17]–[21]. Based on this figure, we support the recommendation of at least *BRCA1* testing for all ovarian cancer patients, regardless of family history and age of diagnosis.

The notably high prevalence of *BRCA1* mutations in our familial patient group (40.6%) places patients in this category (personal history of ovarian cancer and at least one family member with breast and/or ovarian cancer, including personal history of breast cancer) in a high-risk setting, making screening for *BRCA1* mutations mandatory. Previous studies are consistent with this observation and recommendation, reporting *BRCA1* mutation prevalence in hereditary ovarian cancer patients from 24–66% [33]. In accordance with other studies, the age of disease onset was not included in our criteria on selecting familial ovarian cancer patients. Walsh *et al.* [13] have shown that >35% of carriers of a predisposing allele for ovarian cancer are over 60 years of age at diagnosis.

This study also showed that the most common phenotype of *BRCA1*-associated ovarian carcinomas is high-grade serous histology and advanced stage disease. Other histological types were also observed in *BRCA1* carriers, the second most common being the endometrioid type, but there were also clear cell, adenocarcinomas, undifferentiated carcinoma and other types (Tables 4,5). Recent histopathological studies on early stage lesions of ovarian cancer in *BRCA1* or *BRCA2* carriers have suggested that the primary origin of high-grade serous ovarian cancer is the fallopian tube, implicating both genes as key players in the fallopian differentiation [34]–[39]. In our study, 61.5% of the *BRCA1* carriers of the sporadic cases and 78.8% of the familial cases had a serous histology. Further studies on the histology of ovarian cancer subtypes and the molecular events that control the initiation and progression of serous cancers are warranted by a number of research groups.

Nowadays, genetic testing is offered primarily to patients with early onset breast/ovarian cancer (<45 years) or when a strong family history is present, and it primarily involves analysis of *BRCA1* and *BRCA2* genes. Clinical intervention strategies are offered to carriers that will dramatically reduce ovarian cancer risk [33]. The most effective preventive measure today for ovarian and fallopian tube cancer, is salpingo-oophorectomy by the age of 40 years and after completion of child bearing. Unfortunately, ovarian cancer surveillance has not yet been proven effective [40].

In our study, 48.3% of familial patients with breast/ovarian cancer that carry a *BRCA1* mutation have a family history. Furthermore, 45.8% of breast/ovarian cancer patients with inherited *BRCA1* and *BRCA2* mutations do not have a clear family history due to a small family structure, predominance of males in the family and/or paternal inheritance, adoption or non-biological father. *BRCA1* and *BRCA2* mutation carriers in these families are at the same risk level for breast and ovarian cancer as women from high-incidence families. At present, women from such families rarely use genetic services. There is no significant difference between the percentage of *BRCA1* mutation carriers in patients with breast/ovarian cancer and those without a family history. This further supports what is highlighted by all recent mutation prevalence studies, including the present one, that all women with ovarian, fallopian tube, or peritoneal carcinoma should undergo comprehensive genetic testing, regardless of age or family history. However, the screening strategy being employed until now, testing one gene at a time is costly, laborious and time-consuming. Analysing first the most commonly mutated regions in each population, provides a population-specific, cost-effective way for testing hereditary breast/ovarian cancer patients for mutations in predisposing genes. This study provides a fast protocol, for testing all ovarian cancer patients in Greece for mutations in the *BRCA1* gene. Massively parallel sequencing is now being used in order to sequence many genes simultaneously at low cost.

It has now emerged that more ovarian cancer patients carry cancer-predisposing mutations and in more genes than previously appreciated. Recent studies using next-generation sequencing technologies have identified more than 12 low-to-medium penetrance novel susceptibility genes that might predispose for ovarian cancer, but their exact prevalence is still under investigation [13]. Thus, inherited breast/ovarian cancer is genetically highly heterogeneous with respect to both genetic loci and alleles involved.

Furthermore, despite mutations in cancer predisposing genes there are also a number of other molecular aberrations that cause ovarian cancer. It is critical to identify these aberrations as they will lead to the development of novel therapeutic strategies. The Cancer Genome Atlas project has analysed 489 high-grade serous ovarian adenocarcinomas for genomic and epigenomic alterations [32] and identified three microRNA subtypes, four promoter methylation subtypes, four ovarian cancer transcriptional subtypes, and a transcriptional signature associated with survival duration. In contrast to other histological types of ovarian cancer, 96% of high-grade serous (HGS)-OvCa samples had TP53 mutations leading to FOXM1 overexpression and 22% of tumours had germline or somatic mutations in *BRCA1* and *BRCA2.* This study also showed that *BRCA1* and *BRCA2* mutations had a positive impact on survival while BRCA1 epigenetically silenced cases have poorer outcomes.

Identifying individuals with mutations in breast/ovarian cancer susceptibility genes is clinically important as *BRCA1/2* carriers have been shown to have increased sensitivity to the novel poly-ADP-ribose (PARP) inhibitors [41], [42]. It is hoped that this novel treatment will benefit both familial and sporadic ovarian or breast tumours that lack *BRCA1/2* expression. In addition, *BRCA1/2* carriers are also more sensitive to platinum- based chemotherapy [43], [44]. Several studies have also demonstrated improved survival outcomes in carriers versus non-carriers [5], [45], [46].

With the advance and speed of mutation detection strategies nowadays, it is anticipated that more ovarian cancer cases will be avoided as hereditary cancers will be identified before disease onset and preventive measures would be employed.
