# Correction: Prevalence of *BRCA1* Mutations in Familial and Sporadic Greek Ovarian Cancer Cases

**DOI:** 10.1371/annotation/159f0ba3-5a24-4de9-99be-1cd6d5037760

**Published:** 2013-04-17

**Authors:** Alexandra V. Stavropoulou, Florentia Fostira, Maroulio Pertesi, Marianthi Tsitlaidou, Gerassimos E. Voutsinas, Olga Triantafyllidou, Aristotelis Bamias, Meletios A. Dimopoulos, Eleni Timotheadou, Dimitrios Pectasides, Christos Christodoulou, George Klouvas, Christos Papadimitriou, Thomas Makatsoris, George Pentheroudakis, Gerasimos Aravantinos, Vassilis Karydakis, Drakoulis Yannoukakos, George Fountzilas, Irene Konstantopoulou

There was an error in the funding statement. The correct funding information is:

This research has been co-financed by the European Union (European Social Fund – ESF) and Greek national funds through the Operational Program "Education and Lifelong Learning" of the National Strategic Reference Framework (NSRF) - Research Funding Program of the General Secretariat for Research & Technology: ARISTEIA. Investing in knowledge society through the European Social Fund. 

